# Clinical and Functional Assessment of Digenicity in Renal Phosphate Wasting

**DOI:** 10.3390/nu15092081

**Published:** 2023-04-26

**Authors:** Friederike Petzold, Ria Schönauer, Andreas Werner, Jan Halbritter

**Affiliations:** 1 Division of Nephrology, University of Leipzig Medical Center, 04103 Leipzig, Germany; 2 Department of Nephrology, Charité Universitätsmedizin Berlin, 10117 Berlin, Germany; 3 Biosciences Institute, Newcastle University, Newcastle upon Tyne NE1 7RU, UK

**Keywords:** digenicity, hypophosphatemia, NaPi2a, NaPi2b, nephrocalcinosis, nephrolithiasis, NHERF1

## Abstract

Apart from increased fluid intake, patients with kidney stone disease (KSD) due to renal phosphate wasting require specific metaphylaxis. NaPi2a, NaPi2c, and NHERF1 regulate plasma phosphate concentration by reabsorbing phosphate in proximal kidney tubules and have been found altered in monogenic hypophosphatemia with a risk of KSD. In this study, we aimed at assessing the combined genetic alterations impacting NaPi2a, NaPi2c, and NHERF1. Therefore, we screened our hereditary KSD registry for cases of oligo- and digenicity, conducted reverse phenotyping, and undertook functional studies. As a result, we identified three patients from two families with digenic alterations in NaPi2a, NaPi2c, and NHERF1. In family 1, the index patient, who presented with severe renal calcifications and a bone mineralization disorder, carried digenic alterations affecting both NaPi transporter 2a and 2c. Functional analysis confirmed an additive genetic effect. In family 2, the index patient presented with kidney function decline, distinct musculature-related symptoms, and intracellular ATP depletion. Genetically, this individual was found to harbor variants in both NaPi2c and NHERF1 pointing towards genetic interaction. In summary, digenicity and gene dosage are likely to impact the severity of renal phosphate wasting and should be taken into account in terms of metaphylaxis through phosphate substitution.

## 1. Introduction

Kidney stone disease (KSD) represents an increasing health burden, with a lifetime incidence of 11% in men and 7% in women [1]. Furthermore, KSD is associated with an increased risk of chronic kidney disease (CKD) [2] and cardiovascular morbidity [3,4]. Mechanisms of kidney stone formation are dependent on gene–environment interactions, where genetic risk meets nutritional factors. Therefore, KSD is considered a complex systemic disorder requiring a specific diagnostic work-up and adjusted metaphylaxis [3]. 

The most important measure of metaphylaxis is fluid intake, aiming at the urinary dilution of lithogenic salts. In addition, high-risk individuals defined by young age, family history, bilateral or recurrent stones, CKD, and extrarenal manifestations require a specific diagnostic evaluation, potentially resulting in specific metaphylaxis. In particular, in familial cases with nephrocalcinosis (NC), bone mineralization disorders, or sarcopenia, calcium and phosphate levels should be examined in the blood and urine. 

Renal phosphate reabsorption is mainly mediated by two sodium-dependent phosphate cotransporters, NaPi2a and NaPi2c, located in the apical brush border of the proximal tubule. While NaPi2a, encoded by the solute carrier family 34 member 1 (*SLC34A1*), is responsible for 70% of renal phosphate reabsorption [4], NaPi2c (encoded by solute carrier family 34 member 3 (*SLC34A3*)) accounts for the remaining 30% of transport activity [5]. The reabsorption capacity of NaPi transporters is regulated by the level of protein expression, with tightly regulated insertion into the apical membrane and subsequent endocytosis [6]. Additionally, NHERF1 (*NHERF1*, formerly known as *SLC9A3R1*), a sodium/hydrogen exchanger regulatory co-factor, acts as a cytosolic scaffold protein for membrane-associated proteins in the proximal tubule. NHERF1 stabilizes NaPi2a and the parathyroid hormone receptor (PTHR1) and regulates PTH signaling to NaPi2a via cAMP-activating protein kinase A (PKA) [7,8,9] as well as the phospholipase C (PLC)-initiated protein kinase C (PKC) pathway [10]. In addition, interactions between NHERF1 and NaPi2c have been reported, indicating their role in maintaining basal levels of apical NaPi2c expression [11], while the exact mechanisms behind the inducement of NaPi2c endocytosis by PTH coupled to PTHR1 is still unknown. Lastly, PiT-2 (encoded by solute carrier family 20 member 2 (*SLC20A2*)) represents another phosphate transporter protein expressed in the apical membrane of kidney tubules. Its exact contribution to renal phosphate reabsorption, however, has not yet been fully clarified. PiT-2 was found upregulated in NaPi2a-deficient mice during metabolic acidosis [12], and thus may have a compensatory role. To date, genetic alterations have not been associated with renal phosphate loss in humans. Instead, its dysfunction has been shown to be associated with the local hyperphosphatemia-associated vascular calcification of basal ganglia in the central nervous system (idiopathic basal ganglia calcification (IBGC), also known as Fahr’s disease, MIM #213600) [13].

Interestingly, with the exception of PiT-2, the other three players in renal phosphate homeostasis have been found altered in monogenic hypophosphatemia with a risk of consecutive renal calcification and kidney stone formation. Pathogenic variants in *SLC34A1* and *SLC34A3* impair NaPi function and result in urinary phosphate wasting, low serum phosphate levels, increased serum calcitriol levels, and hypercalciuria (MIM #613388 and #241530). Genetic alterations of NHERF1 were shown to increase the PTH-dependent [14] as well as PTH-independent [8] down-regulation of NaPi2a. Therefore, NHERF1 deficiency may indirectly provoke hyperphosphaturic hypophosphatemia with consecutive hypercalciuria, promoting the formation of calcium kidney stones and renal calcification (MIM #612287).

Recently, clinical cases with variants in more than one of the three genes (*SLC34A1*, *SLC34A3*, and *NHERF1*) indicating digenic or oligogenic inheritance have been independently reported in the literature (Supplementary Table S1). However, the mechanistic downstream effect and functional consequences have never been assessed [15,16]. Examples of digenicity in hereditary kidney disease include Alport syndrome caused by pathogenic alterations in more than one of the three *COL4A3-5* genes [17]; cystinuria with variants in both *SLC3A1* and *SLC7A9* [18]; and Bardet–Biedl syndrome, wherein oligogenicity has been postulated for a long time [19].

Theoretically, combined genetic alterations of both *SLC34A1/SLC34A3* and *NHERF1* could either act in an additive way and cause more severe phenotypes or lead to mutual compensation with preserved function [20]. In this study, we aimed at the clinical and molecular characterization of gene–gene interactions by assessing the combined genetic variation of NaPi2a, NaPi2c, and/or NHERF1, the key players in phosphate homeostasis in the human kidney.

## 2. Materials and Methods

### 2.1. Patient Recruitment and Study Protocol

We screened for oligo- and digenicity within the clinical patient Registry of Hereditary Kidney Stone Disease at the University of Leipzig Medical Center, Germany [21]. Patients with kidney stones and/or renal calcifications due to renal phosphate wasting harboring variants in two known disease-causing genes were included in this study. Affected individuals were clinically and metabolically characterized over time (e.g., basic demographics including family history; past medical history; drug history; renal ultrasound; CT scans; X-rays; serum electrolytes; and urinalysis, including urinary calcium/creatinine ratio and urinary pH; blood pH; PTH; vitamin D status; bone scans; and dual-energy X-ray absorptiometry). Whenever available, missing values were filled in through the consultation of electronic health records for the most complete documentation of the course of the disease. 

For genetic testing, genomic DNA was extracted from peripheral blood samples by standard methods. Genetic analyses were performed using next-generation sequencing (NGS)-based panel diagnostics targeting known and candidate genes associated with kidney stone disease (alphabetical order: *ADCY10*, *AGXT*, *ALPL*, *AMMECR1*, *AP2S1*, *APRT*, *ATP6V0A4*, *ATP6V1B1*, *CA2*, *CASR*, *CLCN5*, *CLCNKB*, *CLDN10*, *CLDN16*, *CLDN19*, *CYP24A1*, *FAM20A*, *GDNF*, *GNA11*, *GRHPR*, *HNF4A*, *HOGA1*, *HPRT1*, *KCNJ1*, *MAGED2*, *OCRL*, *OXGR1*, *SLC12A1*, *SLC13A5*, *SLC22A12*, *SLC26A1*, *SLC26A6*, *SLC26A7*, *SLC2A9*, *SLC34A1*, *SLC34A3*, *SLC3A1*, *SLC4A1*, *SLC7A9*, *SLC7A13*, *NHERF1*, *TRPV5*, *TRPV6*, *VDR*, and *XDH*), which was applied to all patients in the registry (patient 1 and patient 2). Additionally, exome sequencing was performed in selected patients or when panel diagnostics remained inconclusive (patient 3). Variant classification was based on published diagnostic criteria of the American College of Medical Genetics and Genomics (ACMG) and compared to given data entries in ClinVar (https://www.ncbi.nlm.nih.gov/clinvar/, accessed on 21 April 2023): class 1—benign; class 2—likely benign; class 3—variant of unknown significance (VUS); class 4—likely pathogenic; class 5—pathogenic [22]. Written informed consent was obtained from all patients upon registry enrollment. Study procedures were approved by the Medical Ethics Committee of the University of Leipzig (Germany) (Institutional Review Board (IRB), University of Leipzig: Ethics vote 159/14-ff).

### 2.2. In Vitro Studies

The in vitro overexpression assay of green fluorescent protein (GFP)-tagged mutant and wild-type NaPi2c [23] and red fluorescent protein (RFP)-tagged mutant and wild-type NaPi2a constructs [24] and the quantification of apical membrane localization in human embryonic kidney cells (HEK293) were performed as follows: HEK293 cells were spread on μ-Slide 8 Wells (ibidi, Martinsried, Germany) and grown to 60–80% confluence in Dulbecco’s modified Eagle’s medium (DMEM, Gibco/Thermo Fisher Scientific, Waltham, USA) supplemented with 10% fetal bovine serum (FBS, S0615; Sigma-Aldrich/Merck, Darmstadt, Germany) under a humidified atmosphere at 37 °C and 5% CO_2_. Cells were transfected with 250 ng plasmid each using Lipofectamine 3000 (Invitrogen/Thermo Fisher Scientific, Waltham, MA, USA) in DMEM without FBS for 3h and then incubated overnight in fresh DMEM + FBS. After 24 h of transfection, the cells were starved in 200 μL Opti-MEMTM Reduced Serum Medium with GlutaMAXTM supplement (Thermo Fisher Scientific, Waltham, MA, USA); the membrane expression of fluorescently labeled proteins in living cells was documented using an AxioObserver Z1 microscope with an ApoTome Imaging System; and images were edited with the ZEN light software (Carl Zeiss AG, Oberkochen, Germany).

The expression of mutated *SLC34A1/3* and *NHERF1* cDNA in *Xenopus laevis* oocytes was assayed as follows: Constructs carrying the relevant mutations were fabricated by overlapping PCR using GoTaq^®^ Long PCR Master Mix (Promega). Starting with the wild-type cDNA, two amplicons were prepared using mutation primers for forward and reverse reactions in combination with a reverse primer over the stop codon extended with a 15nt poly dT stretch and a forward primer over the starting ATG containing a T7 RNA polymerase binding site. Overlapping PCR was then performed with purified, diluted fragments and the poly dT/T7 extended flanking primers. Full-length constructs were column-purified (GeneJet PCR purification Kit, Thermo Fisher Scientific), and 0.5 μg of the amplicons was used for in vitro transcription (mMESSAGE mMACHINE™ T7 Transcription Kit, Thermo Fisher Scientific). 

*Xenopus* oocytes (excised ovarian sacs) were purchased from the European *Xenopus* Resource Centre (EXRC), and established protocols for maintenance and injections were followed [25]. Oocytes were defolliculated as described using Collagenase A (Roche) [25], injected with 10 ng of each of the various constructs, incubated for 4–6 days in modified Barth’s solution, and then assayed for phosphate uptake. Phosphate uptake was performed for 20–30 min with a 0.5 mM carrier concentration and ^33^P (Hartmann Analytics, Germany) as a tracer. Oocytes were dissolved in 1% SDS and counted in a Beckmann scintillation counter. 

## 3. Results

### 3.1. Phenotypic Characterization

**Figure 1:**
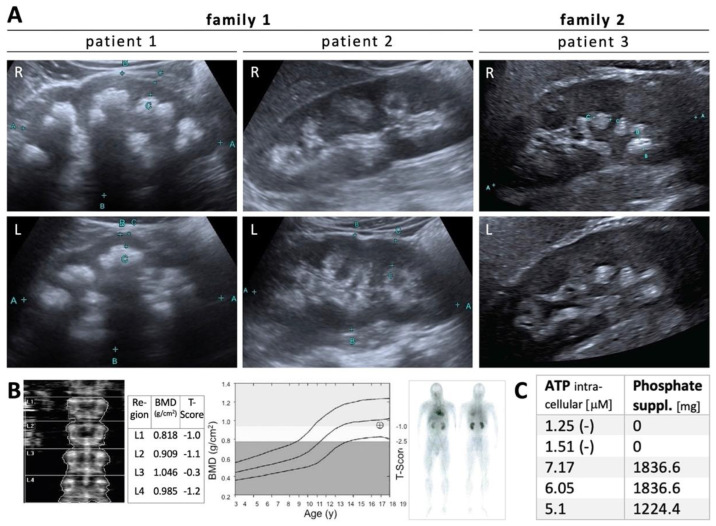
(**A**) Renal ultrasound of family 1 (index patient 1 with severe nephrocalcinosis and his mother, patient 2, with mild kidney calcifications) and family 2 (patient 3 with mild nephrocalcinosis). (**B**) Bone scan and DXA (spinal bone mineral density) of index patient 1. (**C**) Quantitative intracellular ATP analysis (normal range > 2.5 μM) in patient 3 before and after oral phosphate supplementation.

We identified three patients from two non-consanguineous families with digenic variants in the genes *SLC34A1*, *SLC34A3*, and *NHERF1* that were associated with renal phosphate wasting. Upon kidney ultrasound, all of them showed signs of renal calcifications ranging from mild to severe nephrocalcinosis (Figure 1A). 

Since early childhood, patient 1 had experienced severe medullary nephroclacinosis with clusters of calcifications around the renal pyramids as well as several episodes of symptomatic kidney stone disease. Despite normal bone mineral density (dual-energy X-ray absorptiometry of spinal bone), a bone scan demonstrated increased bone metabolism (Figure 1B). At an age of 18 years, he presented with borderline normal kidney function (eGFR according to CKD-EPI: 82 mL/min/1.73 m^2^). At the same time, he was found to suffer from hypophosphatemia due to reduced tubular phosphate reabsorption and consecutive hypercalcemic hypercalciuria (Table 1). Under the initiation of daily oral phosphate supplementation, serum phosphate and calcium levels largely normalized over a period of seven years. In contrast, patient 2, the mother of patient 1, showed only mild medullary kidney calcifications without kidney stones or signs of bone disease. The laboratory assessment of kidney function displayed no decline (eGFR according to CKD-EPI > 90 mL/min/1.73 m^2^), and serum as well as urine electrolytes presented normal ranges (Table 1). However, the FGF-23 level of patient 2 was significantly increased, unlike in the other two patients (patient 1 and patient 3). This was unexpected, as low-to-normal phosphate would normally lead to decreased FGF-23 levels, as regularly seen in patients with genetic alterations of NaPi or NHERF1 [26]. An elevated FGF-23 level can be observed in distinct hereditary disorders including X-linked vitamin D-resistant rickets, as well as in association with cardiovascular disease and bone-related cancer. None of these diseases were diagnosed in patient 2, and no definite cause was eventually assigned to the elevated FGF-23 levels. Nevertheless, patient 2 suffered from anemia due to iron deficiency; for this, an interconnection with FGF-23 has previously been described, whereby low serum iron levels correlate with higher serum FGF-23 [27]. While high FGF-23 C-terminal (cFGF-23) levels may not reflect high intact FGF-23 (iFGF-23), it has been reported that cFGF-23 plasma concentrations increase in women with iron-deficiency anemia [28].

Patient 3 from yet another unrelated family presented with mild nephrocalcinosis without kidney stones but muscular weakness and pain, fatigue, and tachycardia, as well as a stress fracture of the tibia at 31 years of age. Interestingly, severely reduced intracellular ATP levels were documented from peripheral blood cells (Figure 1C). At age 51, her kidney function presented as moderately decreased (eGFR according to CKD-EPI: 56 mL/min/1.73m^2^). Her former medical history revealed marked hypophosphatemia at 0.45 mmol/L as well as hypercalcemic hypercalciuria with decreased tubular phosphate reabsorption (Table 1). Hypophosphatemia, hypercalciuria, and depleted intracellular ATP-levels and musculature-related symptoms recovered under daily phosphate supplementation (Figure 1C).

### 3.2. Genotypic Characterization

**Figure 2:**
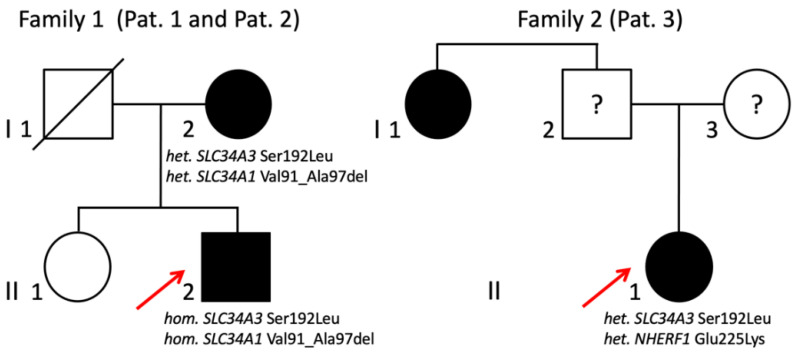
Genetic analysis of two families with multiplex kidney stone disease presenting digenic mutations in *SLC34A1*, *SLC34A3,* and *NHERF1*. Index patient 1 (II2) and patient 2 (his mother, I2) from family 1 with combined genetic variants in NaPi2a and NaPi2c, and index patient 2 from an unrelated family 2 with combined alteration of NaPi2c and NHERF1.

All patients carried a known pathogenic missense variant in *SLC34A3*: c.575C>T, p.(Ser192Leu) (NM_080877.2). While patient 1 was homozygous for this variant, his mother, patient 2, as well as patient 3, showed merely heterozygous alterations. Despite an allele frequency of 0.1% in individuals of European descent (gnomAD, European non-Finnish), this variant was previously shown to significantly reduce phosphate transport activity, resulting in rather mild clinical presentation regarding the severity of nephrocalcinosis and kidney stone disease, most likely depending on gene dosage effects (monoallelic versus biallelic alterations) and other potential factors [23] (Figure 2).

Additionally, NGS-based gene panel diagnostics revealed a common in-frame deletion of seven amino acids at the N terminus of NaPi2a: *SLC34A1* c.272_292del21, p.(Val91_Ala97del) (NM_003052.5). This variant possessed an allele frequency of 2.55% (gnomAD, European non-Finnish) and was found homozygously in patient 1 and heterozygously in his mother, patient 2 (Figure 2). 

The exome sequencing of patient 3 revealed a second heterozygous missense variant in *NHERF1* c.673G>A p.(Glu225Lys) (NM_004252.5) with an allele frequency of 0.0037% (gnomAD, European non-Finnish). Similarly, this genetic alteration was associated with kidney stones and hypophosphatemia due to reduced tubular phosphate reabsorption. Its potentiated PTH-induced inhibition of phosphate uptake compared to wild-type was previously demonstrated by in vitro studies [14] (Figure 2).

### 3.3. Functional Characterization

**Figure 3:**
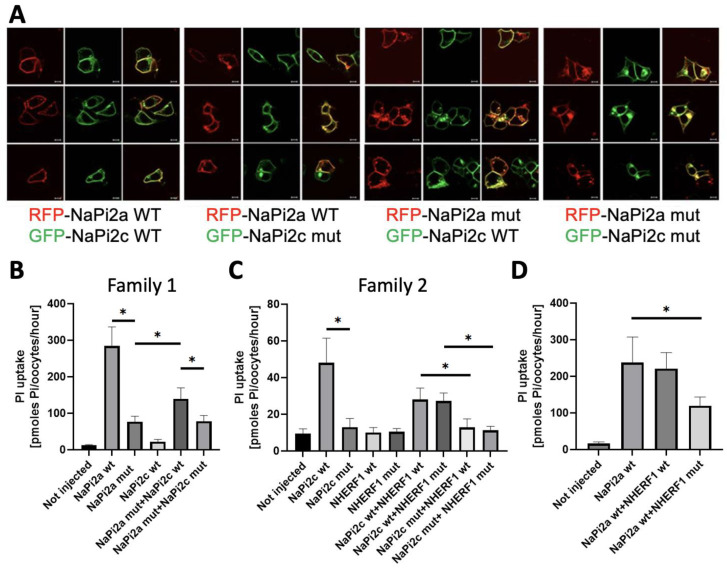
(**A**) Membrane localization of tagged mutant (mut) and wild-type (WT) NaPi constructs in HEK293 cells. Scale bar = 10 µm. (**B**) Phosphate transport activity of combined or individual mutant (mut) and wild-type (WT) NaPi2a and NaPi2c in Xenopus oocytes. (**C**) Phosphate transport activity of combined or individual mutant (mut) and wild-type (WT) NaPi2c and NHERF1 in Xenopus oocytes. (**D**) Phosphate transport activity of NaPi2a-WT alone and in combination with NHERF1-WT versus mutant NHERF1 in *Xenopus* oocytes. * = *p* < 0.05 (significance), Student’s *t*-test.

We next aimed to investigate the functional consequences of the identified germline alterations by in vitro characterization. The combined overexpression of the tagged mutant and wild-type NaPi2a and NaPi2c constructs demonstrated membranous localization in HEK293 cells without significant differences (Figure 3A). However, despite the predominant staining at the cell membrane, mutated transporters also appeared to cluster intracellularly. This trend could be observed with both NaPi2a and NaPi2c mutations, confirming similar previous findings with HEK cells [24] and OK cells [29]. 

To test for potential functional interactions, the mutated NaPi2a and NaPi2c proteins in family 1, NaPi2c and NHERF1 proteins in family 2, and NaPi2a and NHERF1 proteins as a positive control were expressed and assayed for phosphate uptake in *Xenopus* oocytes (Figure 3B–D). In support of potential trafficking defects, the quantification of phosphate uptake upon the injection of wild-type and mutant NaPi in *Xenopus* oocytes showed significantly reduced phosphate transport activity in mutated NaPi2a compared to wild-type, which was partly compensated in combination with wild-type NaPi2c. This compensatory effect was in line with studies that have shown an overexpression of wild-type NaPi2c in NaPi2a-deficient mice [4]. Furthermore, the mutant NaPi2a and aggregated mutant NaPi2a/NaPi2c showed the most reduced phosphate transport activities (Figure 3B).

In the second experimental series, mutated NaPi2c showed reduced phosphate transport activity compared to wild-type (Figure 3C), but in contrast to the compensation of the altered NaPi2a by wild-type NaPi2c, there was no restitution of the mutated NaPi2c by the wild-type NHERF1. In contrast, the additional overexpression of NHERF1 reduced the phosphate uptake of NaPi2c, regardless of whether it was mutated or not (cf. NaPi2c WT vs. NaPi2c WT + NHERF1 WT and NaPi2c WT + NHERF1, respectively). 

In the third experiment, the co-expression of wild-type NHERF1 and NaPi2a did not lead to a significant increase in NaPi2a-generated phosphate uptake, as shown in previous studies [4]. However, the addition of mutated NHERF1 led to a decrease in phosphate uptake, in line with previous in vitro data that pointed to a pathogenic impact of the p.Glu225Lys allele [14] (Figure 3D).

Since the heterozygous NaPi2c variant *SLC34A3* p.(Ser192Leu) alone causes only a very mild clinical phenotype in carriers, we suspected that its combination with the second mutation of the interacting partner NHERF1 would lead to an aggravated reduction in the phosphate reabsorption capacity. Furthermore, we hypothesized that this significant reduction in phosphate uptake would translate into a more severe human phenotype, as displayed by patient 3, who showed markedly reduced serum phosphate levels as well as distinct extrarenal symptoms including an atraumatic bone fracture and longstanding muscular complaints. To our surprise, however, the phosphate uptake experiments showed that NaPi2c and NHERF1 acted independently, and the mutation-induced decrease in phosphate uptake was not significantly affected by the addition of the NHERF1 mutant in our model system (cf. NaPi2c mut vs. NaPi2c mut + NHERF1 WT and NaPi2c mut + NHERF1, respectively) (Figure 3C).

## 4. Discussion

In 7% of adult KSD patients from our in-house registry of hereditary kidney stone disease (n = 236), a diagnostic variant with molecular diagnosis could be identified [30]. Among these, 19% had pathogenic variants in either *SLC34A1*, *SLC34A3,* or *NHERF1*. Therefore, renal phosphate wasting is among the most frequent causes of hereditary KSD. Interestingly, particular NaPi and NHERF variants that were found pathogenic upon functional assessment show a rather high allele frequency in the general population (such as *SLC34A1*-p.Val91_Ala97del, which is present heterozygously in up to 5% of the European population). These variants, which often lead to only mild phosphate wasting, may put individuals at risk rather than mediating completely penetrant kidney stone disease and renal calcification [23]. This is in line with the recent analysis from the UK biobank and FinnGen consortia (comprising 653,219 individuals in total), which found that the common *SLC34A1* in-frame deletion (p.Val91_Ala97del) is associated with an increased risk of kidney stone disease in the general population [31]. However, in a recently published *Slc34a1* knock-in mouse model with mono- or biallelic Val91_Ala97 deletion, no signs of impaired phosphate homeostasis were identified [32]. Nevertheless, such high allele frequencies contribute to the likelihood of coincident findings of two or more variants in phosphate transporters and/or co-factors, as this was also the case in 1.3% of the individuals in our kidney stone disease registry cohort (3/236).

**Figure 4:**
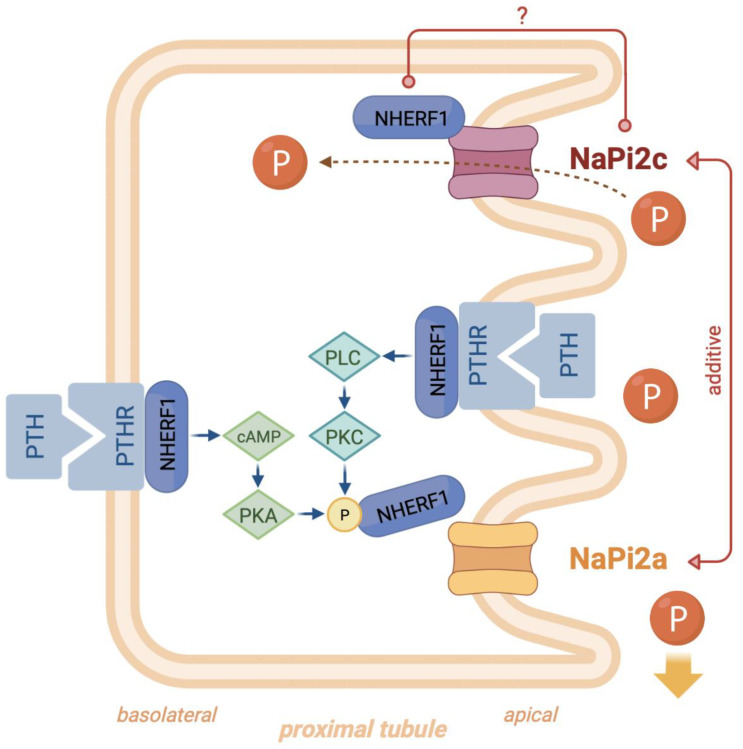
Pathogenic variants of NaPi2a/2c and NHERF1 proteins disrupt renal phosphate reabsorption and cause urinary phosphate wasting. The co-factor NHERF1 acts as a scaffold protein stabilizing NaPi2a and the parathyroid hormone receptor (PTHR), regulating PTH signaling to NaPi2a via cAMP-activating protein kinase A (PKA) as well as the phospholipase C (PLC)-initiated protein kinase C (PKC) pathway [33]; both of these lead to the phosphorylation of NHERF1 to uncouple it from NaPi2a and inhibit phosphate uptake. Therefore, genetic defects in NHERF1 increase PTH-induced cAMP generation but also functional alterations between the direct interaction of NHERF1 and NaPi2a, both resulting in an enhanced inhibition of phosphate uptake and urinary phosphate wasting. NHERF1 interacts with NaPi2a by classical PDZ-binding via a sequence located at the C-terminus of the protein. This C-terminal binding motif is changed in NaPi2c, and therefore NHERF1 interaction and regulation differs from NaPi2a. While NaPi2a transport activity depends directly on the presence of NHERF1 [9], our data suggested an additive effect upon NaPi2a and NaPi2c dysfunction; a phenomenon that we could not observe for simultaneous alterations in both NaPi2c and NHERF1.

NaPi2a and NaPi2c constitute the major renal players in regulating the plasma concentration by reabsorbing phosphate from the proximal tubule (Figure 4). In line with a previous clinical report [15], our data suggested that NaPi2a and NaPi2c act in concert, as the genetic dysfunction of one renal phosphate transporter may in part be compensated by the other transporter, resulting in attenuated phenotypes [23]. In the absence of this compensation due to digenic or oligogenic defects in both phosphate transporters, phosphate reabsorption activity was significantly reduced, leading to more severe presentations of renal calcification and bone mineralization disorders, as seen in patient 1.

In vitro and in vivo studies have characterized the interaction between NaPi2a and NHERF1 in depth, demonstrating that the phosphate transport capacity of NaPi2a depends directly on the expression level of NHERF1 [9,34,35]. In detail, previous studies demonstrated that the co-expression of NaPi2a with wild-type NHERF1 enhanced phosphate uptake compared to NaPi2a alone, whereas the combined expression of mutant NHERF1 and NaPi2a had similar results to what was observed for NaPi2a alone in the absence of NHERF1 [8]. Although our studies did not demonstrate such an additional effect of wild-type NHERF1, which was possibly due to a dosing effect as oocytes express intrinsic NHERF1, the addition of mutated NHERF1 did result in a significant decrease in phosphate uptake.

Less is known about the interaction between NaPi2c and NHERF1 [11]. In contrast to the additive effect seen upon the mutation of both NaPi2a and 2c, our data suggested that NaPi2c and NHERF1 act rather independently. Nevertheless, a limitation of our study was the absence of PTH as a stimulating factor in NHERF1-mediated phosphate wasting. Therefore, we could not fully exclude an additive effect upon simultaneous defects in both NHERF1 and NaPi2c based on our model. Anyhow, the clinical presentation of the moderate-to-severe phenotype with kidney function decline as well as muscle weakness and pain (patient 3) was more severe than is usually seen in monoallelic NaPi2c conditions and did at least suggest genetic interaction.

Further limitations of our study concern the use of non-polarized HEK293 cells for overexpression, as we were unable to detect any trafficking defects. Opossum kidney cells (OK cells), as a more suitable model of proximal tubular cells expressing both NaPi2a and PTH type 1 receptor (PTH1R), would have better reflected the physiological conditions of phosphate homeostasis.

NHERF1 consists of two tandem postsynaptic density 95/disc large/zona occludens (PDZ) domains [36]. The detected variant in patient 3 affected the second PDZ domain (PDZ2) of the NHERF1 protein (Figure 4), which was reported to interact with the PTH receptor (PTHR1) by increasing PTH binding and cAMP generation [31]. In vitro studies in human cell lines and *Xenopus* oocytes showed the consistent impairment of apical sorting but ambiguous effects on the transport capacity of mutant cells with reduced phosphate transport activity [24] or no clear transport defect [29]. The enhanced phosphorylation of NHERF1 leads to uncoupling from the NaPi2a transporter with consecutive urinary phosphate loss [7,14]. The NaPi2a C-terminus interacts with NHERF1 through classical PDZ-binding involving an -ATRL motif (Figure 4). This C-terminal binding site is not conserved between the two isoforms, and therefore the NaPi2c–NHERF1 interaction differs from NaPi2a [7]. There are implications that the putative NaPi2c–NHERF1 interaction is essential for maintaining a basal level of NaPi2c apical expression but is not required to adapt to dietary phosphate uptake [11]. Therefore, we hypothesized that the combined genetic alteration of *NHERF1* and *NaPi2c* would impact predominantly basal phosphate levels resulting in decreased muscle ATP synthesis, muscle weakness and pain, and cardiac symptoms, as seen in patient 3 [37].

The molecular and clinical diagnosis of renal phosphate loss led to the initiation of phosphate supplementation in patient 1 and 3. Under regular intake and adjusted dosing based on serum phosphate and calcium levels, further kidney calcification, stone formation, and extrarenal symptoms can be attenuated. In children, the correction of chronic hypophosphatemia enables normal growth and prevents ricket formation. 

However, oral phosphate supplementation may have some side effects, such as diarrhea, which occurs mostly at the start of therapy and can be influenced by adjusting the administration time and dosage. Long-term oral therapy requires the monitoring of serum phosphate calcium, 1,25-dihydroxyvitamin D levels, and PTH, which is important to prevent hyperparathyroidism and nephrocalcinosis [38].

In summary, our report, as well as recently published case studies [15,16], on patients carrying additional variants in *SLC34A1*, *SLC34A3*, and *NHERF1* suggested that oligo- and digenicity play a role in disorders of renal phosphate wasting (Supplementary Table S1). The co-occurrence of relatively common but intermediate-effect-size alleles, such as *SLC34A1* in-frame deletion and other *SLC34A3* variants, is likely underdiagnosed and should be considered upon complete genetic evaluation. Due to gene dosage effects and genetic interaction, oligo- and digenicity is likely to result in a more severe clinical presentation. However, the type and localization of the variant also contribute to the expressivity and phenotypic presentation. Our study illustrated that, in addition to the general recommendation of increased fluid intake, a stratified work-up of kidney stone disease patients is essential to inform specific metaphylaxis.

## Figures and Tables

**Table 1:** Clinical and initial biological presentation of three patients with kidney stones and renal calcifications due to digenicity-caused renal phosphate wasting. Normal ranges: eGFR/CKD-EPI > 90 mL/min/1.73 m^2^; serum phosphate 0.84–1.45 mmol/L; FGF-23 26–110 RU/mL; TrP (tubular reabsorption of phosphate) 82–90%; TmP/GFR (tubular maximum reabsorption of phosphate/glomerular filtration rate ratio) 0.84–1.23; serum calcium 2.15–2.50 mmol/L; urine calcium < 0.57 mmol/mmol Crea; serum PTH 1.6–6.9 pmol/L; 1.25 (OH)_2_ vitamin D_3_ 15.2–90.1 pg/mL; 25(OH) vit. D3 > 20 ng/mL. LP, likely pathogenic; P, pathogenic; VUS, variant of uncertain significance; (−) decreased value/below normal range; (+) increased value/above normal range.

	Fam 1, Pat 1	Fam 1, Pat 2	Fam 2, Pat 3
**Nephrocalcinosis/kidney stone disease**	Severe nephrocalcinosis, recurrent kidney stones	Mild nephrocalcinosis	Mild nephrocalcinosis
**Bone disease**	Increased bone metabolism, short stature	No	Stress fracture tibia
**Other symptoms**	No	No	Muscle weakness/pain, tachycardia
**Phosphate supplement**	Yes	No	Yes
**Sex**	Male	Female	Female
**Age** (time of phenotype exploration)	18	47	51
**Digenic variants,** ACMG classification	- Hom. ***SLC34A3*** p.(Ser192Leu); P (PS3, PM1, PM2, PP5) - Hom. ***SLC34A1*** p.(Val91_Ala97del); VUS (PS3, BS1)	- Het. ***SLC34A3*** p.(Ser192Leu); P (PS3, PM1,PM2,PP5) - Het. ***SLC34A1*** p.(Val91_Ala97del); VUS (PS3,BS1)	- Het. ***SLC34A3*** p.(Ser192Leu); P (PS3, PM1,PM2,PP5) - Het. ***NHERF1*** p.(Glu225Lys); LP (PS3, PM1, BP4)
**eGFR** (mL/min/1.73 m^2^)	82 (−)	100	56 (−)
**Serum phosphate** (mmol/L)	0.63 (−)	1.11	0.45 (−)
**FGF-23** C-terminal (RU/mL)	36	835 (+)	62
**TrP** (%)	77 (−)	88	68.5 (−)
**TmP/GFR** (mmol/L)	0.49 (−)	0.98	0.69 (−)
**Serum calcium** (mmol/L)	2.68 (+)	2.40	2.51
**Urine calcium** (Ca/Crea ratio in mmol/mmol Crea)	0.23	0.54	0.23
**Serum PTH** (pmol/L)	0.80 (−)	2.12	2.88
**1.25 (OH)_2_ vit. D_3_** (pg/mL)	44.0	70.7	54.8
**25(OH) vit. D_3_** (ng/mL)	17.2 (−)	25.8	24.6

## Data Availability

The data of this study are available from the corresponding authors upon request.

## Supplementary Materials

The following supporting information can be downloaded at: https://www.mdpi.com/article/10.3390/nu15092081/s1, Table S1: Previous reported case series of patients carrying digenic or oligogenic variants in genes associated with kidney stone disease due to renal phosphate wasting including their genotype and kidney/extrarenal phenotype.

## References

[1] Scales C.D., Smith A.C., Hanley J.M., Saigal C.S. (2012). Prevalence of Kidney Stones in the United States. Eur. Urol..

[2] Meneses J.A., Lucas F.M., Assunção F.C., Castro J.P.P., Monteiro R.B. (2012). The Impact of Metaphylaxis of Kidney Stone Disease in the Renal Function at Long Term in Active Kidney Stone Formers Patients. Urol. Res..

[3] Zeng G., Zhu W., Robertson W.G., Penniston K.L., Smith D., Pozdzik A., Tefik T., Prezioso D., Pearle M.S., Chew B.H. (2022). International Alliance of Urolithiasis (IAU) Guidelines on the Metabolic Evaluation and Medical Management of Urolithiasis. Urolithiasis.

[4] Tenenhouse H.S., Martel J., Gauthier C., Segawa H., Miyamoto K. (2003). Differential Effects of *Npt2a* Gene Ablation and X-Linked *Hyp* Mutation on Renal Expression of Npt2c. Am. J. Physiol.-Renal Physiol..

[5] Ohkido I., Segawa H., Yanagida R., Nakamura M., Miyamoto K. (2003). Cloning, Gene Structure and Dietary Regulation of the Type-IIc Na/Pi Cotransporter in the Mouse Kidney. Pflug. Arch.

[6] Lötscher M., Kaissling B., Biber J., Murer H., Levi M. (1997). Role of Microtubules in the Rapid Regulation of Renal Phosphate Transport in Response to Acute Alterations in Dietary Phosphate Content. J. Clin. Investig..

[7] Mahon M.J., Donowitz M., Yun C.C., Segre G.V. (2002). Na+/H+ Exchanger Regulatory Factor 2 Directs Parathyroid Hormone 1 Receptor Signalling. Nature.

[8] Courbebaisse M., Leroy C., Bakouh N., Salaün C., Beck L., Grandchamp B., Planelles G., Hall R.A., Friedlander G., Prié D. (2012). A New Human NHERF1 Mutation Decreases Renal Phosphate Transporter NPT2a Expression by a PTH-Independent Mechanism. PLoS ONE.

[9] Weinman E.J., Cunningham R., Wade J.B., Shenolikar S. (2005). The Role of NHERF-1 in the Regulation of Renal Proximal Tubule Sodium-Hydrogen Exchanger 3 and Sodium-Dependent Phosphate Cotransporter 2a: NHERF-1 and Regulation of Renal Proximal Tubule NHE3 and Npt2a. J. Physiol..

[10] Capuano P., Bacic D., Roos M., Gisler S.M., Stange G., Biber J., Kaissling B., Weinman E.J., Shenolikar S., Wagner C.A. (2007). Defective Coupling of Apical PTH Receptors to Phospholipase C Prevents Internalization of the Na+-Phosphate Cotransporter NaPi-IIa in Nherf1-Deficient Mice. Am. J. Physiol.-Cell Physiol..

[11] Villa-Bellosta R., Barac-Nieto M., Breusegem S.Y., Barry N.P., Levi M., Sorribas V. (2008). Interactions of the Growth-Related, Type IIc Renal Sodium/Phosphate Cotransporter with PDZ Proteins. Kidney Int..

[12] Nowik M., Picard N., Stange G., Capuano P., Tenenhouse H.S., Biber J., Murer H., Wagner C.A. (2008). Renal Phosphaturia during Metabolic Acidosis Revisited: Molecular Mechanisms for Decreased Renal Phosphate Reabsorption. Pflug. Arch..

[13] Zhang Y., Guo X., Wu A. (2013). Association between a Novel Mutation in SLC20A2 and Familial Idiopathic Basal Ganglia Calcification. PLoS ONE.

[14] Karim Z., Gérard B., Bakouh N., Alili R., Leroy C., Beck L., Silve C., Planelles G., Urena-Torres P., Grandchamp B. (2008). *NHERF1* Mutations and Responsiveness of Renal Parathyroid Hormone. N. Engl. J. Med..

[15] Gordon R.J., Li D., Doyle D., Zaritsky J., Levine M.A. (2020). Digenic Heterozygous Mutations in SLC34A3 and SLC34A1 Cause Dominant Hypophosphatemic Rickets with Hypercalciuria. J. Clin. Endocrinol. Metab..

[16] Brazier F., Courbebaisse M., David A., Bergerat D., Leroy C., Lindner M., Maruani G., Saint Jacques C., Letavernier E., Hureaux M. (2023). Relationship between Clinical Phenotype and in Vitro Analysis of 13 NPT2c/SCL34A3 Mutants. Sci. Rep..

[17] Mencarelli M.A., Heidet L., Storey H., van Geel M., Knebelmann B., Fallerini C., Miglietti N., Antonucci M.F., Cetta F., Sayer J.A. (2015). Evidence of Digenic Inheritance in Alport Syndrome. J. Med. Genet..

[18] Font-Llitjós M., Jiménez-Vidal M., Bisceglia L., Di Perna M., de Sanctis L., Rousaud F., Zelante L., Palacín M., Nunes V. (2005). New Insights into Cystinuria: 40 New Mutations, Genotype-Phenotype Correlation, and Digenic Inheritance Causing Partial Phenotype. J. Med. Genet..

[19] Perea-Romero I., Solarat C., Blanco-Kelly F., Sanchez-Navarro I., Bea-Mascato B., Martin-Salazar E., Lorda-Sanchez I., Swafiri S.T., Avila-Fernandez A., Martin-Merida I. (2022). Allelic Overload and Its Clinical Modifier Effect in Bardet-Biedl Syndrome. NPJ Genom. Med..

[20] Schäffer A.A. (2013). Digenic Inheritance in Medical Genetics. J. Med. Genet..

[21] Halbritter J., Seidel A., Müller L., Schönauer R., Hoppe B. (2018). Update on Hereditary Kidney Stone Disease and Introduction of a New Clinical Patient Registry in Germany. Front. Pediatr..

[22] Richards S., Aziz N., Bale S., Bick D., Das S., Gastier-Foster J., Grody W.W., Hegde M., Lyon E., Spector E. (2015). Standards and Guidelines for the Interpretation of Sequence Variants: A Joint Consensus Recommendation of the American College of Medical Genetics and Genomics and the Association for Molecular Pathology. Genet. Med..

[23] Schönauer R., Petzold F., Lucinescu W., Seidel A., Müller L., Neuber S., Bergmann C., Sayer J.A., Werner A., Halbritter J. (2019). Evaluating Pathogenicity of SLC34A3-Ser192Leu, a Frequent European Missense Variant in Disorders of Renal Phosphate Wasting. Urolithiasis.

[24] Fearn A., Allison B., Rice S.J., Edwards N., Halbritter J., Bourgeois S., Pastor-Arroyo E.M., Hildebrandt F., Tasic V., Wagner C.A. (2018). Clinical, Biochemical, and Pathophysiological Analysis of *SLC34A1* Mutations. Physiol. Rep..

[25] Markovich D. (2008). Expression Cloning and Radiotracer Uptakes in Xenopus Laevis Oocytes. Nat. Protoc..

[26] Alizadeh Naderi A.S., Reilly R.F. (2010). Hereditary Disorders of Renal Phosphate Wasting. Nat. Rev. Nephrol..

[27] Edmonston D., Wolf M. (2020). FGF23 at the Crossroads of Phosphate, Iron Economy and Erythropoiesis. Nat. Rev. Nephrol..

[28] Wolf M., Rubin J., Achebe M., Econs M.J., Peacock M., Imel E.A., Thomsen L.L., Carpenter T.O., Weber T., Brandenburg V. (2020). Effects of Iron Isomaltoside vs Ferric Carboxymaltose on Hypophosphatemia in Iron-Deficiency Anemia: Two Randomized Clinical Trials. JAMA.

[29] Schlingmann K.P., Ruminska J., Kaufmann M., Dursun I., Patti M., Kranz B., Pronicka E., Ciara E., Akcay T., Bulus D. (2016). Autosomal-Recessive Mutations in *SLC34A1* Encoding Sodium-Phosphate Cotransporter 2A Cause Idiopathic Infantile Hypercalcemia. JASN.

[30] Schönauer R., Scherer L., Nemitz-Kliemchen M., Hagemann T., Hantmann E., Seidel A., Müller L., Kehr S., Voigt C., Stolzenburg J.-U. (2022). Systematic Assessment of Monogenic Etiology in Adult-Onset Kidney Stone Formers Undergoing Urological Intervention-Evidence for Genetic Pretest Probability. Am. J. Med. Genet. C Semin. Med. Genet..

[31] Sun B.B., Kurki M.I., Foley C.N., Mechakra A., Chen C.-Y., Marshall E., Wilk J.B., Chahine M., Chevalier P., Biogen Biobank Team (2022). Genetic Associations of Protein-Coding Variants in Human Disease. Nature.

[32] Bieri C., Daryadel A., Bettoni C., Pastor-Arroyo E.-M., Schnitzbauer U., Hernando N., Wagner C.A. (2022). The Human Pathogenic 91del7 Mutation in SLC34A1 Has No Effect in Mineral Homeostasis in Mice. Sci. Rep..

[33] Mamonova T., Friedman P.A. (2021). Noncanonical Sequences Involving NHERF1 Interaction with NPT2A Govern Hormone-Regulated Phosphate Transport: Binding Outside the Box. Int. J. Mol. Sci..

[34] Hernando N., Gisler S.M., Pribanic S., Déliot N., Capuano P., Wagner C.A., Moe O.W., Biber J., Murer H. (2005). NaPi-IIa and Interacting Partners. J. Physiol..

[35] Shenolikar S., Voltz J.W., Minkoff C.M., Wade J.B., Weinman E.J. (2002). Targeted Disruption of the Mouse NHERF-1 Gene Promotes Internalization of Proximal Tubule Sodium-Phosphate Cotransporter Type IIa and Renal Phosphate Wasting. Proc. Natl. Acad. Sci. USA.

[36] Mamonova T., Kurnikova M., Friedman P.A. (2012). Structural Basis for NHERF1 PDZ Domain Binding. Biochemistry.

[37] Pesta D.H., Tsirigotis D.N., Befroy D.E., Caballero D., Jurczak M.J., Rahimi Y., Cline G.W., Dufour S., Birkenfeld A.L., Rothman D.L. (2016). Hypophosphatemia Promotes Lower Rates of Muscle ATP Synthesis. FASEB J..

[38] Felsenfeld A.J., Levine B.S. (2012). Approach to Treatment of Hypophosphatemia. Am. J. Kidney Dis..

