# Supplementary material for: Clinical and Functional Assessment of Digenicity in Renal Phosphate Wasting
Source: Nutrients. 2023 Apr 26;15(9):2081. doi: 10.3390/nu15092081 (PMC10180814; doi:10.3390/nu15092081)
Supplement: Supplementary file 1 [file nutrients-15-02081-s001.zip › nutrients-2345107-supplementary.pdf]

ID	Genes	Variants (ACMG classification)	Kidney phenotype	Extrarenal phenotype	Laboratory analysis	Ref.
III-7	***SLC34A3*** (het)	*c.1561dupC, p.Leu521Profs*72* (LP)	bilateral kidney stones	short stature, rickets	hypophosphatemia, hypercalciuria	[15]
	SLC34A1 (het)	*c.1535G>A, p.Arg512His* (VUS)				
III-6	***SLC34A3*** (het)	*c.1561dupC, p.Leu521Profs*72* (LP)	bilateral kidney stones	short stature, rickets	hypophosphatemia	[15]
	SLC34A1 (het)	*c.1535G>A, p.Arg512His* (VUS)				
II-5	***SLC34A3*** (het)	*c.1561dupC, p.Leu521Profs*72* (LP)	no	rickets, short stature	normal serum phosphate	[15]
	SLC34A1 (het)	*c.1535G>A, p.Arg512His* (VUS)				
2	***SLC34A3*** (het)	*c.496G>A, p.Gly166Ser* (P)	recurrent nephrolithiasis	no	hypophosphatemia, CKD G2	[16]
	SLC34A3 (het)	*c.1093+41_1094-15del, p.?* (LP)				
	SLC34A1 (het)	*c.272_292del, p.Val91_Ala97del* (VUS)				
4	***SLC34A3*** **(**het)	*c.496G>A, p.Gly166Ser* (P)	recurrent nephrolithiasis	short stature	hypophosphatemia, hypercalciuria, CKD G1	[16]
	SLC34A1 (het)	*c.1416+3G>A, p.?* (VUS)				
	NHERF1 (het)	*c.328C>G, p.Leu110Val* (LP)				
6	***SLC34A3*** (hom)	*c.575C>T, p.Ser192Leu* (LP)	recurrent nephrolithiasis	mineralization disorder	hypophosphatemia, CKD G2	[16]
	NHERF1 (het)	*c.328C>G, p.Leu110Val* (LP)				
8	***SLC34A3*** (het)	*c.781A>G, p.Ser261Gly* (LB)	mild	no	hypophosphatemia	[16]
	SLC34A1 (het)	*c.644+5G>A, p.?* (VUS)				

Supplementary Table S1: Previous reported case series of patients carrying digenic or oligogenic variants in genes associated with kidney stone disease due to renal phosphate wasting including their genotype and kidney/extrarenal phenotype.

Abbreviations: het heterozygous; hom homozygous; LB likely benign; VUS variant of unknown significance; LP likely pathogenic; P pathogenic; CKD chronic kidney disease; CKD stages (G) according KDIGO classification); Ref. Reference.
